# Effects of a Dehydroevodiamine-Derivative on Synaptic Destabilization and Memory Impairment in the 5xFAD, Alzheimer's Disease Mouse Model

**DOI:** 10.3389/fnbeh.2018.00273

**Published:** 2018-11-13

**Authors:** Shinwoo Kang, Sungji Ha, Hyunjun Park, Eunjoo Nam, Won Hyuk Suh, Yoo-Hun Suh, Keun-A Chang

**Affiliations:** ^1^Department of Pharmacology, College of Medicine Gachon University, Incheon, South Korea; ^2^Neuroscience Research Institute Gachon University, Incheon, South Korea; ^3^Department of Health Sciences and Technology, GAIHST Gachon University, Incheon, South Korea; ^4^Department of Bioengineering, College of Engineering, Temple University Philadelphia, PA, United States

**Keywords:** Alzheimer's disease, carboxy-dehydroevodiamine, memory impairment, 5xFAD, synaptic destabilization

## Abstract

Carboxy-dehydroevodiamine·HCl (cx-DHED) is a derivative of DHED, which improves memory impairment. Carboxyl modification increases solubility in water, indicating that its bioavailability is higher than that of DHED. Cx-DHED is expected to have better therapeutic effects on Alzheimer's disease (AD) than DHED. In this study, we investigated the therapeutic effects of cx-DHED and the underlying mechanism in 5xFAD mice, transgenic (Tg) mouse model of AD model mice. In several behavioral tests, such as Y-maze, passive avoidance, and Morris water maze test, memory deficits improved significantly in cx-DHED-treated transgenic (Tg) mice compared with vehicle-treated Tg mice. We also found that AD-related pathologies, including amyloid plaque deposition and tau phosphorylation, were reduced after the treatment of Tg mice with cx-DHED. We determined the levels of synaptic proteins, such as GluN1, GluN2A, GluN2B, PSD-95 and Rabphilin3A, and Rab3A in the brains of mice of each group and found that GluN2A and PSD-95 were significantly increased in the brains of cx-DHED-treated Tg mice when compared with the brains of Tg-vehicle mice. These results suggest that cx-DHED has therapeutic effects on 5xFAD, AD model mice through the improvement of synaptic stabilization.

## Introduction

Alzheimer's disease (AD), a common neurodegenerative disease, is characterized by amyloid beta (Aβ) plaque and intracellular neurofibrillary tangles (NFTs) (Cortese and Burger, 2016). Other important pathologies of AD are neuroinflammation (Daulatzai, 2016) and synaptic dysfunction (La Joie et al., 2014). The progression of AD reveals symptoms, such as language problems, disorientation, and memory impairment (Van Cauwenberghe et al., 2016). Approximately 4,000 new cases of AD are diagnosed in the world per day, and 28–33 million people worldwide suffer from AD (Wortmann, 2012). By 2020, an estimated 43.2 million people worldwide will suffer from AD (Ferri et al., 2005). The costs of caring for patients with AD are increasing annually, which imposes a tremendous financial and social burden on the community and patients' families.

Currently, drugs used for the treatment of AD only bring some degree of symptomatic relief but fail to cure or delay disease progression (Selkoe, 2011). Several studies with regard to inhibiting Aβ aggregation have been reported (Gervais et al., 2001; Bilikiewicz and Gaus, 2004; Aisen et al., 2006; Townsend et al., 2006; Santa-Maria et al., 2007; Tohda, 2016), but no drug has been identified that effectively blocks or reverses the process of AD progression until now. During the last decade, all phase III clinical trials including promising candidates, such as Solanezumab and Verubecestat have failed because of adverse effects and the lack of cognitive improvement (Egan et al., 2018; Honig et al., 2018).

Dehydroevodiamine·HCl (DHED) is a component purified from *Evodia rutaecarpa* Bentham (Park et al., 1996). In previous studies, DHED improved spatial memory deficit by inhibiting acetylcholinesterase (Decker, 2005; Unsworth et al., 2013) and modulating the activity of glycogen synthase kinase-3 (Peng et al., 2007). Furthermore, DHED prevented impairments of learning and memory in a scopolamine-induced memory impairment rat model (Park et al., 1996, 2000) and had beneficial effects on memory impairment and depressive-like behavior in a stressed rat model (Kim et al., 2014b). Despite these well-studied effects of DHED on cognition, the underlying mechanism of these effects of DHED remains unknown. Carboxy-DHED (cx-DHED; cx-DD, Supplementary Figure 1), a derivative of DHED, is highly soluble in water, because of this it is expected to have higher bioavailability and better therapeutic effects on AD than DHED.

The 5xFAD AD mice model represents a very aggressive amyloid deposition that exhibits accumulation of intraneuronal Aβ_42_ at 1.5 months, amyloid plaques at 2 months, memory deficits at 4 months, and neuronal loss at 9 months of age (Oakley et al., 2006). In addition, 5xFAD mice developed age-dependent behavioral deficits when studied using Y-maze, passive avoidance test, and Morris water maze test (Kim et al., 2014a; Webster et al., 2014). These characteristics make 5xFAD mice a robust model for investigating cx-DD's pharmaceutical potential for the treatment of AD. In this study, we investigated the therapeutic effects of cx-DD on memory loss from 4 months of age in 5xFAD mice and its underlying mechanisms.

## Materials and methods

### Animals

The 5xFAD transgenic (Tg) mice, which express five human APP and PS1 genes [three in APP (Swedish mutation: K670N, M671L; Florida mutation: I716V; London mutation: V717I) and two in PS1 (M146L, L286V)], at 4 months of age were used. A 12L: 12D photoperiod was provided, and temperature and humidity of the breeding room were automatically maintained at 22 ± 2°C and 50 ± 10%, respectively. Food and water were provided *ad libitum* during the acclimation period to the polycarbonate cage. All animal experiments were approved by the Institutional Animal Care and Use Committee of the Lee Gil Ya Cancer and Diabetes Institute, Gachon University (LCDI-2015-0025).

### Experimental procedures

**Figure 1:**
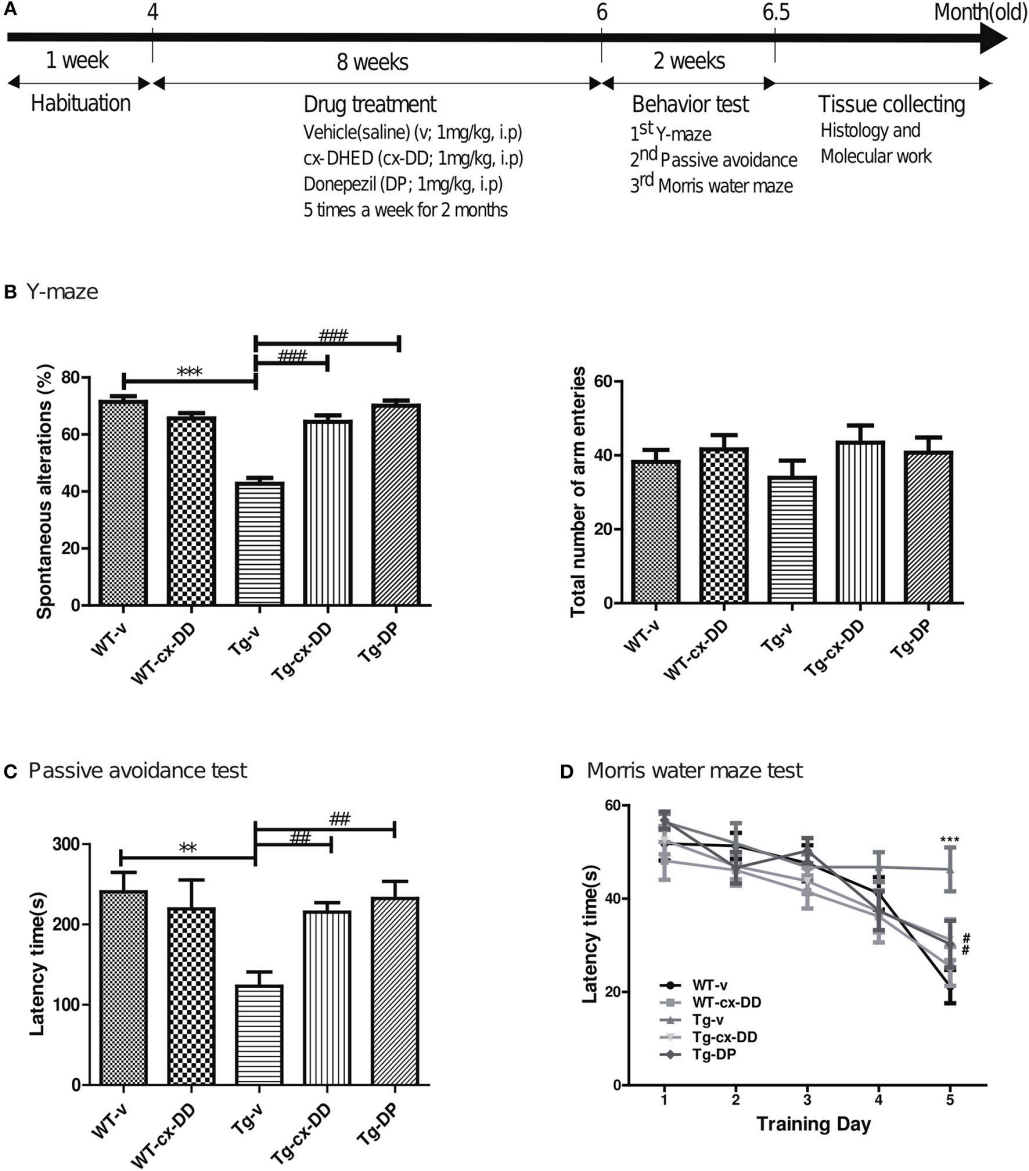
Schematic timeline of drug treatment and order of behavioral tests in 5xFAD mice and wild-type (WT) control mice and the effects of cx-DHED on learning and memory deficits in 5xFAD mice during behavioral training trials. **(A)** The 4-months 5xFAD transgenic male mice were tested after 2 months of five times weekly cx-DHED (cx-DD), donepezil (DP), or vehicle (v) injections after 1 week of habituation in the animal room. At the end of the tests, all mice were decapitated, and their brains were collected. **(B)** The percentage of spontaneous change increased significantly in response to cx-DD and DP compared with that in Tg mice (left panel). No difference in the number of arm entries was detected between the experimental groups (right panel). **(C)** When mice entered the dark chamber, an electrical foot shock (0.2 mA) was delivered for 2 s. Latency increased significantly in the mice treated with cx-DD and DP compared with that in the untreated Tg mice. **(D)** The training trial was carried out four times daily for five consecutive days. The treatments with Cx-DD and DP significantly decreased latency in Tg mice. All data were given as means ± standard error of the mean (SEM) (*N* = 10 mice per group). **P<*0.01*, ***P<*0.001* compared with WT-v mice, ^#^*P*<*0.05*, ^##^*P*<*0.01*, ^###^*P*<*0.001* compared with Tg-v mice by ANOVA. Vehicle-treated wild type mice (WT-v); cx-DHED-treated wild type mice(WT-cx-DD); vehicle-treated 5xFAD Tg mice (Tg-v); cx-DHED-treated 5xFAD Tg mice(Tg-cx-DD); DP-treated 5xFAD Tg mice (Tg-DP).

In each experimental group, 4-month old Tg or wild type (WT) mice were used and consisted of 10 male mice per group, which are as follows: vehicle-treated wild type mice (WT-v); cx-DHED-treated wild type mice (WT-cx-DD); vehicle-treated 5xFAD Tg mice (Tg-v); cx-DHED-treated 5xFAD Tg mice (Tg-cx-DD); and Donepezil (DP)-treated 5xFAD Tg mice (Tg-DP) (Figure 1A).

Carboxy-DD was suspended in 0.9% saline solution and administered at a dose of 1 mg/kg by intraperitoneal (i.p.) injection five times a week for 2 months before behavior tests, and the 0.9% saline solution in the same volume was administered to the control group. Donepezil (1 mg/kg), which is a clinically used drug for AD, was dissolved in saline and administered to Tg-DP group as positive control. When the animals were 6 months old, behavioral tests were conducted to determine changes in cognition level. The day after the behavioral test, brains were collected from the animals (Figure 1A).

### Behavior tests

We conducted three behavioral tests with five groups of 5xFAD mice to assess changes in cognition and memory, and the mice were rested for 1 day between each behavioral test. The Y-maze task consisted of three branches (A, B, and C) that were 40-cm long, 5-cm wide, and 10-cm high at an angle of 120°. The maze was constructed of white polyvinyl plastic. The animal was placed in the maze for 8 min, and the frequency that the tail entered each branch was counted for each branch. The number of times the animal entered the branches (in A, B, C sequence) was also counted and awarded one point (real change, actual alternation). Ability to take action to change (%) was calculated using the following formula: actual change (actual alternation)/maximum change (maximum alternation) × 100 (maximum change: the total entrance number – 2).

The passive avoidance task was performed using the avoidance learning box (Gemini Passive Avoidance System; San Diego Instruments, San Diego, CA, USA). The passive avoidance apparatus (42.5-cm wide and 35.5-cm long) consisted of a two-chamber box with lighted and darkened compartments. The light part was illuminated by a LED house light (6 W) and connected to the dark chamber, which was equipped with an electric grid floor. The animals were allowed to explore both the compartments on the first day. On the following day, the entrance of the animals to the dark box was punished by an electric shock to the feet (2 mA for 2 s). This training procedure was carried out between 9:00 a.m. and 2:00 p.m. Retention test session was performed 24 h after training and was procedurally similar to training except that no shock was given to the mice. The time duration for which the mouse remained in the bright room (step-through latency) was recorded as a measure of memory retrieval.

Spatial reference memory was evaluated using the Morris water maze (MWM) test. Mice were trained to learn to use spatial cues to locate a hidden platform in a circular pool (diameter 140 cm, height 45 cm, outer height [from the ground] 61.5 cm) filled with opaque water, which is equilibrated to room temperature (22°C). The pool area was subdivided into four virtual quadrants that were defined as target quadrants, containing the goal platform (Supplementary Figure 3A). During the test, escape latency, swim path (distance), speed (velocity), as well as quadrant preference, was recorded using the EthoVision Maze task system (Noldus Information Technology, Wageningen, the Netherlands). The animals underwent four training trials per day (one time per quadrant) over five consecutive days with a 30-min interval. If the animal could not find the platform within 60 s, they were placed on the platform for 20 s. After removal from the pool, mice were manually dried with a terrycloth towel and placed in a warming cage (consisting of a heating pad set to low underneath a typical cage) for at least 5 min before returning to the home cage. Mice were visually inspected to ensure thorough dryness. On the seventh day, a probe test was administered. During the probe test, the platform was removed from the pool and the mouse was allowed to swim freely for 1 min. After the probe test, all tracks from all trials were analyzed for some behavioral parameters using the software from the EthoVision Maze task system. The resultant behavioral data were statistically analyzed as described below.

### Tissue preparation

The mice were anesthetized with Rompun and Zoletil (1 μg/g, i.p.) and transcardially perfused with cold saline containing heparin after conducting the behavioral tests. Brains were divided into two hemispheres for immunohistochemistry and western blotting.

One hemisphere was immediately deep frozen by dissecting into cortex (CX) and hippocampus or without dissection. For whole brain lysates, frozen whole hemispheres of 6-months-old 5xFAD mice (*n* = 4–5 per group) were weighed and homogenized in RIPA buffer containing a cocktail of protease inhibitors (Roche Science, Mannheim, Germany) and a cocktail of phosphatase inhibitors (Sigma Aldrich, St. Louis, MO, USA). For the fraction of synaptosome, frozen cortical or hippocampal tissues of 6-months-old 5xFAD mice (*n* = 4–5 per group) were weighed and extracted by adding a synaptic protein extraction reagent (87793, Thermo scientific).

The other hemisphere was fixed overnight in 4% paraformaldehyde at 4°C and embedded in paraffin. Brains were sectioned coronally on a microtome of 4-μm thickness. Serial sections were placed on a slide glass.

### LC-MS/MS analysis of cx-DHED

Samples were analyzed using an Agilent LC-1200 series combined with an Agilent 6410 triple-quadrupole mass spectrometer (MS) (Agilent Technologies, USA) system. A chromatographic method was developed using a YMC-Pack Pro C8 column (4.6 × 150 mm, 3 μm, YMC, Japan). The column oven was maintained at 40°C. The following gradient elution with 5 mM ammonium formate in water as “A” and 5 mM ammonium formate in methanol as “B” was used at a flow rate of 600 μL/min: 0 min, 20% B; 0–8 min, 20 → 65% B; 8–9 min, 65 → 95% B; 9–14 min, 95% B; 14–14.5 min, 95 → 20% B; 14.5–22 min, 20% B. The injection volume was 20 μL. Mass spectrometric analysis was performed in the positive mode with an electrospray ionization (ESI) source. In scan mode, scan range was 50–500 *m*/*z*, and the parameter settings for ESI were as follows: nebulizer, 45 psi; gas flow, 10 L/min; gas temperature, 300°C; capillary voltage, 4 kV. Quantification analysis was performed using the multiple reaction monitoring (MRM) mode by applying the parameters shown in Supplementary Table 1.

### Immunohistochemistry

Coronal sections of 4 μm-thickness on a slide glass were deparaffinized and hydrated. Antigen was retrieved in 0.01 M citric acid at 56°C and non-specific binding of antibodies was blocked using blocking buffer (0.5% Triton-X with 20 μl/ml goat serum in PBS) three times for 5 min each, followed by a 4°C overnight incubation with primary antibodies to 6E10 (1:1,000, 39320, Sigma-Aldrich) and AT8 (1:1,000, MN1020, Thermo Scientific, Waltham, MA, USA). The Vectastain Universal Elite ABC Kit (PK-6102, Vector Laboratories, Burlingame, CA, USA) was used as the secondary antibody and incubated for 1 h at room temperature.

Extracellular Aβ load and paired helical filaments (PHFs)-tau were evaluated in CX and dentate gyrus (DG) of the hippocampus, using a Zeiss AxioImager Z1 microscope equipped with an Axiocam HRC camera and the ImageJ software (V1.4.3.67, NIH, USA). Serial images of 40 × or 100 × magnification were captured on an average of 2–3 sections per animal. The Aβ plaque load and the amount of PHFs-tau in the same brain region of the same size were measured with blind count and presented as numbers in the area.

### Western blot analysis

Proteins in whole brain lysates or synaptosome fraction were quantified using the BCA protein assay kit (Thermo Scientific) subjected to sodium dodecyl sulfate-polyacrylamide gel electrophoresis under reducing conditions. The proteins were transferred onto a polyvinylidene difluoride membrane in transfer buffer, incubated for 1 h in blocking solution (6% skim milk) at room temperature, and incubated with GluN2A (1:1,000, PA5-27921, Invitrogen, Carlsbad, CA, USA), PSD-95 (1:3,000, MA1-046, Invitrogen), Rph3A (1:1,000, AB59259, Abcam, Cambridge, MA, USA), RAB3A (1:1,000, WH0005864M1, Sigma, St. Louis, USA), AT8 (1:1,000, MN1020, Thermo Scientific, Waltham, MA, USA), H150 (1:300, SC-5587, Santa Cruz Biotechnology, Dallas, USA), GAPDH (1:3,000, AP0066, Bioworld Biotechnology, St. Louis Park, MN, USA), and beta-actin (1:2,000, SC-47778, Santa Cruz Biotechnology, Dallas, USA) primary antibodies at 4°C overnight. The next day, the membrane was washed three times with Tris-buffered saline with Tween 20 (TBS-T) and incubated with a horseradish peroxidase-conjugated secondary antibody for 1 h at room temperature. After three washes in TBS-T, the membrane was visualized by an enhanced chemiluminescent PicoEPD Western Reagent kit (ELPIS-Biotech., Inc, Seoul, Korea). Immunoblots were imaged using the BLUE detection medical X-ray film (AGFA, Mortsel, Belgium). Quantification of the blots was carried out using the Image J software.

### Statistical analysis

Differences between groups were tested with either one-way or two-way analysis of variance (ANOVA) followed by the Newman-Keuls *post-hoc* test. All data were given as means ± standard error of the mean (SEM). All calculations were performed using the GraphPad Prism software (GraphPad Software Inc.).

## Results

### Cx-DHED restored cognitive and memory deficits of a 5xFAD mouse model

To test whether cx-DHED (cx-DD) has therapeutic effects on cognitive impairment of AD, we first evaluated cognitive behavior performance of five groups (WT-v; WT-cx-DD; Tg-v; Tg-cx-DD; Tg-DP) of mice at 6 months of age. First, all groups of mice were evaluated for the effect of cx-DD treatment on spatial and procedural working memories in the Y-maze. The rate of spontaneous changes in the Y-maze test was significantly improved in Tg-cx-DD (63.19 ± 2.40) or Tg-DP mice (68.72 ± 2.10) compared with Tg-v mice (45.85 ± 2.71) (Figure 1B, left panel). There was no difference in the total number of arm entries in the experimental groups (Figure 1B, right panel). To investigate the effect of cx-DD treatment on short-term reference memory, the passive avoidance test was performed. Step-through latency in Tg-cx-DD (215.4 ± 11.78) or Tg-DP mice (232.4 ± 21.26) was decreased compared with that in Tg-v mice (123.0 ± 17.95) (Figure 1C). To investigate if cx-DD treatment affects hippocampus-related spatial reference and working memory, mice were tested in the MWM. In an acquisition training, Tg-v mice (43.04 ± 3.45) showed deficits compared with WT-v mice (19.11 ± 3.925, *p* < 0.001) on the fifth day (Figure 1D), although the speed of swimming (velocity) did not differ (Supplementary Figure 3B). The Tg-cx-DD (35.65 ± 3.25, *p* < 0.01) mice, as well as the Tg-DP mice (26.98 ± 3.48, *p* < 0.05), showed a significant decrease of escape latency compared with the Tg-v mice (43.04 ± 3.45) on the fifth day (Figure 1D). In a probe test, Tg-v mice showed less preference for the target quadrant compared with those of other groups (Supplementary Figure 3C), although the swimming distance did not differ (Supplementary Figure 3D). The Tg-cx-DD mice traveled into the target quadrant more times than the Tg-v mice, but it was not significant (Supplementary Figure 3C).

These results show that memory deficits significantly improved in the Tg-cx-DD mice compared with the Tg-v mice based on Y-maze, passive avoidance, and MWM test results.

### Cx-DHED penetrated across the blood-brain barrier

To determine whether cx-DD could penetrate the blood-brain barrier (BBB), we performed liquid chromatography (LC)-MS/MS analysis with brain samples of cx-DD injected 5xFAD mice. At 24 h after single injection of 1 mg/kg of cx-DD, a portion of the injected cx-DD was detected in the brain sample (0.0109 ng/mg protein ± 0.0001, *n* = 3). A certain amount of cx-DD (0.0166 ng/mg protein ± 0.001, *n* = 3) was also detected in the brain samples from 5xFAD mice daily dosed with 1 mg/kg of cx-DD for 8 weeks. These results suggest that cx-DD could be delivered into the brain through the BBB.

### Cx-DHED reduced the numbers of Aβ plaque

**Figure 2:**
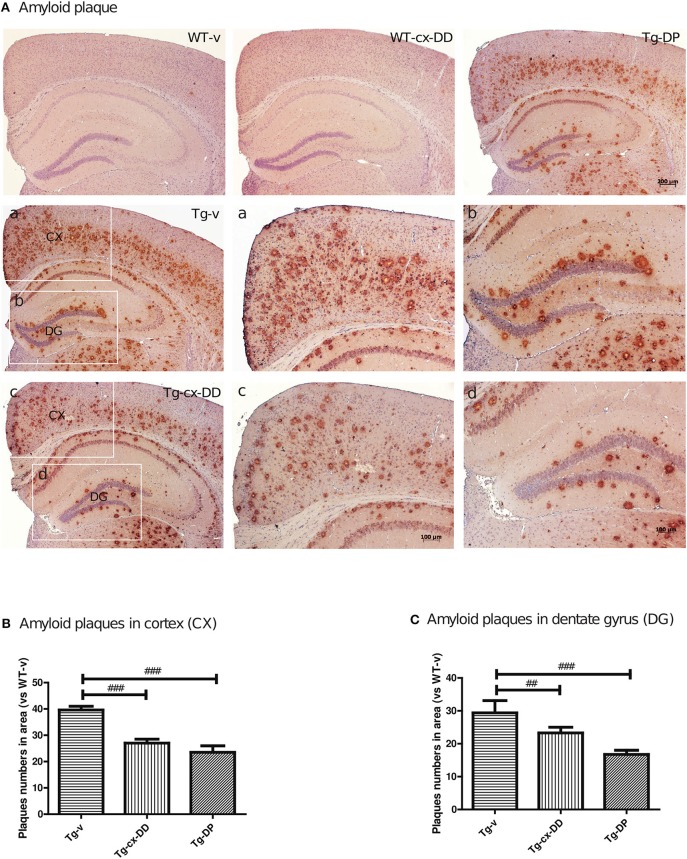
Effects of cx-DHED on amyloid beta (Aβ) plaque deposition. **(A)** Immunostaining of brain tissues with the 6E10 antibody from WT-v, WT-cx-DD, Tg-v, Tg-cx-DD, and Tg-DP groups. Scale bars, 40 × . a–d: the cortex (a,c) and dentate gyrus (b,d) of the hippocampal region are enlarged images of square box in Tg-v and Tg-cx-DD, scale bars, 100 × . **(B,C)** Plaque counts in the cortex (CX; **B**) and dentate gyrus (DG; **C**) of the hippocampal region decreased in Tg-cx-DD mice. All data were given as means ± standard error of the mean (SEM) (*N* = 7 mice per group). ^##^*P*<*0.01*, ^###^*P*<*0.001* compared with Tg-v mice.

To investigate whether the cx-DD-mediated improvement in cognitive function impairments was due to changes in the level of Aβ, we assayed the level of Aβ by immunohistochemistry with a specific antibody against Aβ (6E10 antibody). The number of Aβ plaques were counted including intraneuronal Aβ and extracellular plaques and compared with that in CX and DG of the hippocampus of the brains of mice from all the groups (Figure 2). Neuritic plaques were identified in 5xFAD Tg mice, but no plaque was detected in WT mice. The number of Aβ plaques significantly decreased in CX and DG in response to cx-DD or cx-DP treatments compared with that in Tg-v mice (Figure 2A). As shown in Figure 2B, cx-DD (27.0 ± 1.53, *p* < 0.001) or DP (23.5 ± 1.40, *p* < 0.001) treatments significantly decreased the number of Aβ plaques in the CX of Tg-v mice (36.6 ± 1.40). In the DG, the number of Aβ plaques of Tg-cx-DD (23.29 ± 1.742, *p* < 0.05) or Tg-DP mice (16.75 ± 1.25, *p* < 0.001) were significantly decreased compared with that in Tg-v mice (29.4 ± 3.71).

### Cx-DHED reduced tau-related AD pathologies including phosphorylated tau

**Figure 3:**
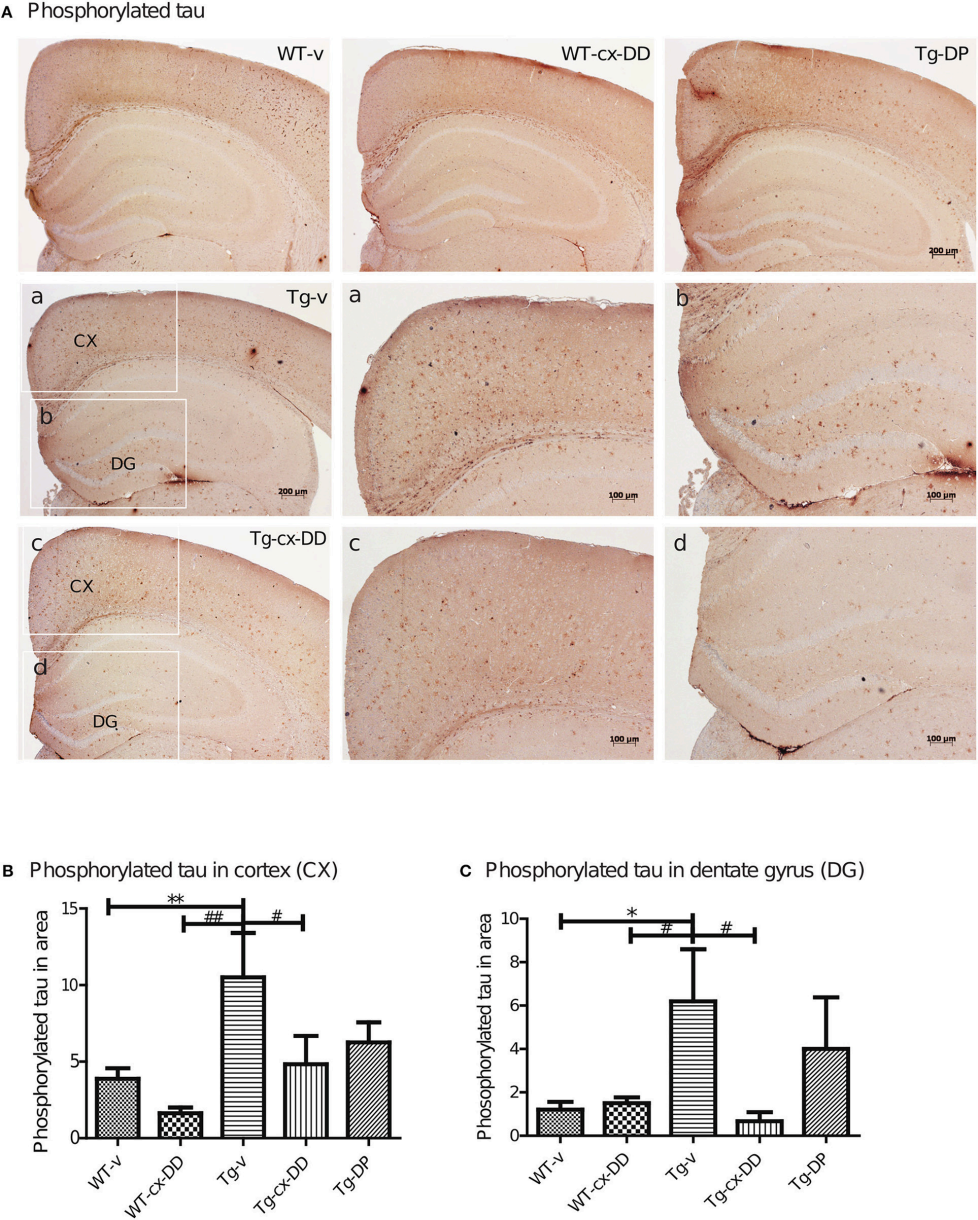
Effects of cx-DHED on phosphorylated tau deposits. **(A)** Immunostained brain tissues with the AT-8 antibody in WT-v, WT-cx-DD, Tg-v, Tg-cx-DD, and Tg-DP. Scale bars, 40 × . a–d: the cortex (a,c) and dentate gyrus (b,d) of the hippocampal region are enlarged images of square box in Tg-v and Tg-cx-DD, scale bars, 100 × . **(B,C)** Phosphorylated tau counts in CX **(B)** and DG **(C)** of the hippocampal region decreased in Tg-cx-DD mice. Scale bars, 100 × . All data were given as means ± standard error of the mean (SEM) (*N* = 4 mice per group). *P<*0.05*, **P<*0.01* compared with WT-v mice, ^#^*P*<*0.05*, ^##^*P*<*0.01* compared with Tg-v mice.

Abnormal folding of tau protein leads to the generation of PHFs and NFTs, one of the key neuropathological features in AD. It has been proposed that extracellular tau aggregates contribute to the propagation of neurodegenerative disease pathogenesis (Frost et al., 2009; Guo and Lee, 2011). We investigated the level of PHFs-tau in the brains of 6 months-old Tg mice by immunohistochemistry with the AT8 specific p-tau antibody. As shown in Figure 3A, Tg-v mice showed increased levels of PHFs-tau in the CX compared with the DG of WT-v mice. However, Tg-cx-DD mice showed significantly decreased PHFs-tau in CX (10.5 ± 2.90 to 4.83 ± 2.27, *p* < 0.05, Figure 3B) and DG (6.2 ± 2.68 to 0.67 ± 0.52, *p* < 0.05, Figure 3C) compared with vehicle treatment. Donepezil treatment also decreased PHFs in CX (10.5 ± 2.90 to 6.25 ± 2.9, ns) and DG (6.2 ± 2.39 to 4.00 ± 2.38, ns) compared with Tg-v, but there was no significance (Figures 3B,C). In whole brain lysates, the amount of PHFs-tau proteins of Tg-v mice significantly increased (5-folds of WT-v, *p* < 0.5) and cx-DD treatment significantly decreased these amounts to control level (Supplementary Figure 2). Amount of PHFs-tau proteins in Tg-DP mice decreased to half the level of those in Tg-v mice (Supplementary Figure 2).

### Changed levels of GluN2A/PSD-95 in synaptosomes

**Figure 4:**
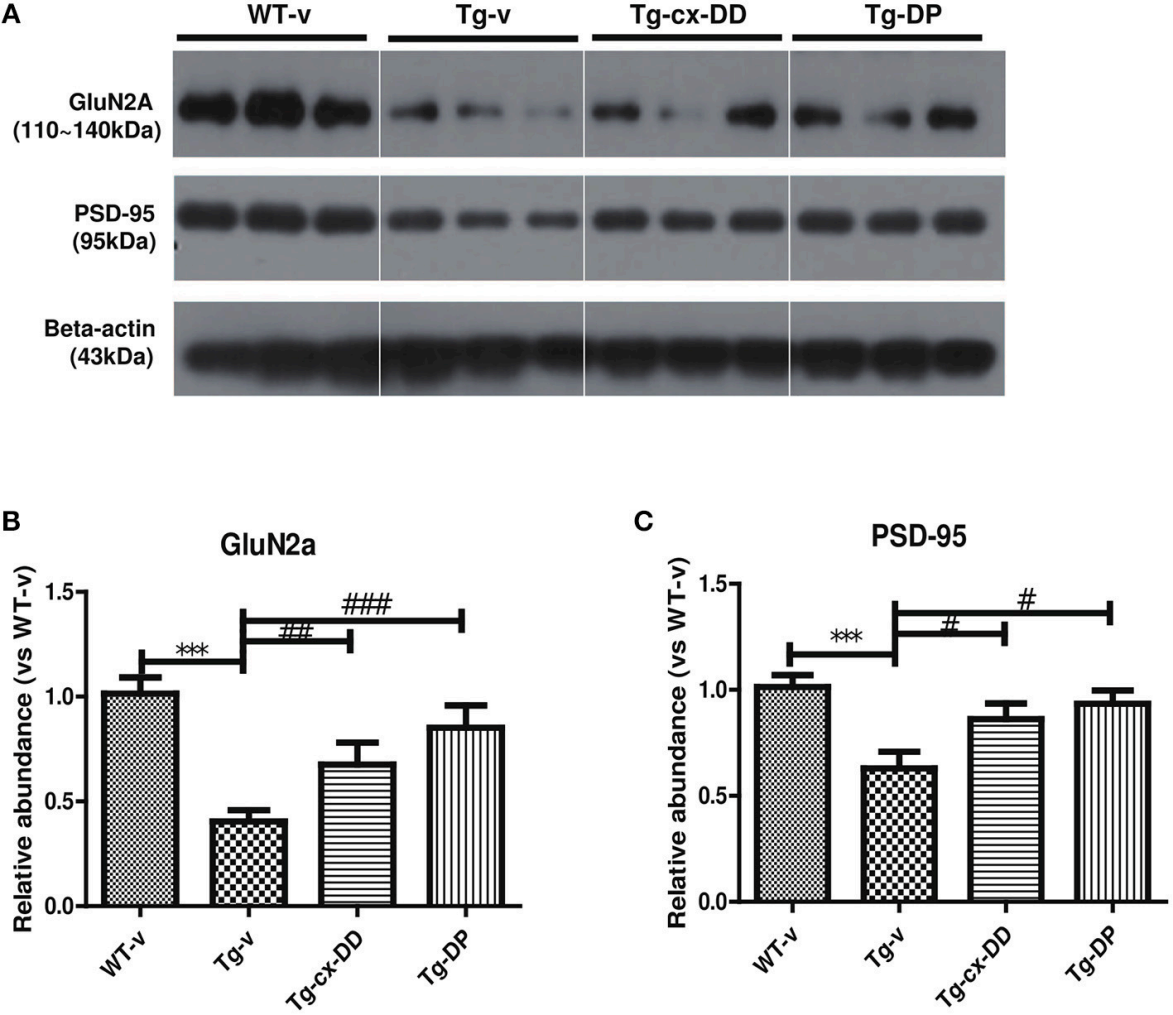
Effects of cx-DHED on synaptic proteins. Synaptosome fractions of cortical lysates were used to detect the loss of post-synaptic receptor immunoreactivity in mice synaptic proteins. **(A)** Western blot analysis of GluN2A, PSD-95, Rabphilin-3A, and RAB3A from cortex tissue lysate. **(B–C)** The bar shows the percentage of beta-actin normalized to the density of GluN2A **(B)** and PSD-95 **(C)**, on Western blot bands from cortex tissue lysate. All data were given as means ± standard error of the mean (SEM) (*N* = 4 mice per group). ***P<*0.001* compared with WT-v mice, ^#^*P*<*0.05*, ^##^*P*<*0.01*, ^###^*P*<*0.001* compared with Tg-v mice.

Next, we focused on the correlation between *p*-tau and synaptic dysfunction. Additionally, PSD-95 is an important factor that contributes to synaptic formation and has been proposed to be a molecular scaffold for receptors and the cytoskeleton in synapses (Cho et al., 1992). N-methyl-D-aspartate receptors (NMDARs) are required for memory acquisition and consolidation (Parsons and Raymond, 2014). In addition, NMDARs are heterotetramers with two GluN1 subunits and two variable subunits, such as GluN2 and GluN3 (Parsons and Raymond, 2014). In the CNS of an adult, GluN2A is a majorly regulated subunit (Paoletti et al., 2013; Sanz-Clemente et al., 2013). We determined the levels of GluN2A and PSD-95 proteins in cortical synaptosome fractions of brains of 6-months-old Tg and age-matched WT mice. As shown in Figure 4, GluN2A expression in the brains of Tg mice decreased to 0.404-fold (*p* < 0.001) compared with that in WT mice. However, expression of GluN2A in the brains of Tg-cx-DD and Tg-DP mice increased to 1.67-fold (*p* < 0.01) and to 2.11-fold (*p* < 0.001), respectively, compared with that in the brains of Tg-v mice (Figure 4B). The low PSD-95 levels in the brains of Tg mice recovered in the brains of Tg-cx-DD (0.86 ± 0.09, *p* < 0.05) and Tg-DP (0.93 ± 0.08, *p* < 0.05) mice (Figure 4C). We also checked the level of other NMDAR subtypes, such as GluN1 and GluN2B by western blotting, but no significant differences were observed between the animal groups (Supplementary Figure 4).

Recently, it was identified that GluN2A-containing NMDARs are stabilized at the synaptic membrane by forming a ternary complex with PSD-95 and Rabphilin-3A (Rph3A) (Stanic et al., 2015). Rph3A is a putative target protein for Ras-associated binding (RAB) 3A small GTP-binding protein (Stanic et al., 2015). Next, we investigated the levels of Rph3A and RAB3A in the same tissues, but the changes in Rph3A and RAB3A were not significant (Supplementary Figure 4).

## Discussion

AD is the most common cause of dementia related to a progressive neurodegenerative disability, with a prevalence in 44 million people worldwide in 2015, and is estimated to double by 2050 (Van Cauwenberghe et al., 2016). Therefore, new therapeutic drugs for AD with fewer side effects and a better effect are urgently needed.

AD is pathologically characterized by Aβ and NFTs. Soluble oligomeric Aβ has been detected in the brain tissues of patients with AD (Giuffrida et al., 2009) and demonstrated to be associated with memory and cognitive impairment (Shankar et al., 2008; Wilcox et al., 2011; Rijal Upadhaya et al., 2012). Recent reports suggested that the ratios of Aβ1–42/Aβ1–40 measured in the cerebrospinal fluid (CSF) are strongly associated with the amount of cortical deposition of Aβ fibrils, leading to improvement in the accuracy of biological conclusions in the diagnosis of patients with AD (Kuperstein et al., 2010; Sauvee et al., 2014; Pannee et al., 2016).

In a previous study, it was reported that DHED is transported from the systemic circulation to the brain via the BBB by linear kinetics (Ahn et al., 2004). Pretreated DHED showed neuroprotective effects against neuronal cell toxicity induced by Aβ1–42 (10 μM) treatment (Kim et al., 2014b). In addition, pretreatment with DHED for 4 months before Aβ1–42 (25 μM) treatment significantly reduced cell death and production of reactive oxygen species (Shin et al., 2017). Moreover, DHED treatment increased memory and cognitive functions in scopolamine-induced memory impairment rat model or ischemic rat model (middle cerebral artery-occluded model; Park et al., 2000).

Based on these results of previous studies, we investigated the therapeutic potential of cx-DHED (cx-DD, Supplementary Figure 1), a derivative of DHED, in a 5xFAD AD animal model. cx-DD is highly soluble in water, because of which it is expected to have higher bioavailability and better therapeutic effects on AD than DHED. We checked if cx-DD could penetrate the BBB by LC-MS/MS analysis. Later, a certain amount of cx-DD was detected in brain samples of cx-DD injected 5xFAD mice. These results show that cx-DD can be delivered to the brain via the BBB.

Our report is the first study to investigate the effects of cx-DD on memory impairments and synaptic instability in the 5xFAD AD mouse model. Interestingly, mice receiving cx-DD treatment recovered their impaired memory, as shown by Y-maze, passive avoidance, and MWM test results (Figure 1). These results demonstrate that cx-DD has therapeutic effects on memory impairment.

To study the underlying mechanisms of improved learning and memory ability induced by cx-DD treatment, we investigated the effect of cx-DD on AD-related pathology. First, we assayed the level of Aβ by immunohistochemistry in WT and Tg mice treated with vehicle or cx-DD. The 6E10 antibody showed strong immunoreactivity across CX and DG of the hippocampus in the brain tissues of 6-months-old Tg mice (Figure 2). Amyloid plaque levels decreased in the group treated with cx-DD (Figure 2). These results suggest that cx-DD might be effective for the reduction of amyloid plaques.

Deposition of Aβ is thought to be due to abnormal tau phosphorylation, which leads to the generation of PHFs and NFTs, one of the key neuropathological features in AD (Hardy and Allsop, 1991). In addition, extracellular tau aggregates contribute to the propagation of neurodegenerative disease pathogenesis (Frost et al., 2009; Guo and Lee, 2011). Therefore, we measured PHFs-tau levels using an AT-8 antibody in CX and DG brain regions of all groups of mice. The levels of PHFs-tau significantly decreased in Tg-cx-DD mice and Tg-DP mice compared with Tg-v mice. Although DP treatments also reduced the level of PHFs-tau in CX and DG regions of brains of Tg mice, the effects of cx-DD treatment were higher than those of DP in the whole brain (Supplementary Figure 2). The levels of PHFs-tau with cx-DD treatment significantly reduced compared with Tg-v mice. In particular, cx-DD reduced the levels of PHF-s tau much more significantly in CX and DG of 6 months-old 5xFAD mice than DP, although cx-DD administration decreased the amounts of amyloid plaque to an extent similar to DP.

Tau hyper-phosphorylation tends to weakening of the tau-microtubule interaction and disrupts neuronal networks. Thus, we hypothesized that the levels of synaptic proteins might decrease in the brains of 5xFAD mice. To demonstrate the effects of cx-DD on synaptic stability, we focused hippocampal NMDARs, which are required for memory acquisition and consolidation. In addition, NMDAR is a heteromeric complex that interacts with intracellular proteins by three different subunits: GluN1, GluN2, and GluN3. In regions of the CNS involved in cognitive functions, like the hippocampus and the prefrontal cortex (PFC), GluN2A, and GluN2B are the major regulatory subunits (Paoletti et al., 2013; Sanz-Clemente et al., 2013). Whereas, GluN2B is predominant in the early post-natal brain. There is an increase in GluN2A expression in the early post-natal period, while GluN2B expression remains constant (Sans et al., 2000). As a result, the ratio of GluN2A/GluN2B increases during this period (Monyer et al., 1994; Hoffmann et al., 2000). Grosshans et al. (2002) showed that after plasticity induction, GluN1 and GluN2A subunits increased in the synaptic fraction (Grosshans et al., 2002). During memory consolidation, GluN1 and GluN2A NMDAR subunits increase in the hippocampus (Cercato et al., 2016). Therefore, we examined the levels of expression of GluN1, GluN2A, GluN2B, and PSD-95 in the brain and found that the levels of GluN2A and PSD-95 were decreased in 5xFAD mice (Figure 4), but the levels of GluN1 and GluN2B were unchanged (Supplementary Figure 4). Together with the reduction in PHFs-tau protein, the protein levels of GluN2A and PSD-95 recovered to control levels in response to cx-DD treatment. Accordingly, cx-DD may have therapeutic potential for synaptic destabilization associated with AD.

Another previous study showed that Rph3A expression is highly reduced in the CX of patients with AD, and Rph3A forms a complex with GluN2A and PSD-95 (Tan et al., 2010; Stanic et al., 2015). Therefore, we hypothesized that disruption of the GluN2A/Rph3A/PSD-95 complex might promote internalization of GluN2A, leading to synaptic dysfunction. However, we found that the levels of Rph3A and Rab3A were not different in all groups of mice (Supplementary Figure 4). However, more synaptic proteins were densely distributed in cortical synaptosome fractions of brains of Tg-cx-DD mice than those in Tg-v mice (Figure 4). These results show that cx-DD attenuated cognitive deficits by increasing synaptic-related proteins, such as GluN2A-containing NMDARs and PSD-95.

In conclusion, we, for the first time, report that cx-DD strongly reduces memory impairment, the number of amyloid plaques, and PHFs-tau, as well as synaptic destabilization in 5xFAD AD animal model mice. These results clearly show that cx-DD could block the progression of AD pathology and memory deficits in 5xFAD mice.

## Author contributions

K-AC and Y-HS supervised the project and designed the experiments. SK and SH carried out the experiments, analyzed the data, and wrote the manuscript. WHS checked purity and stability of the cx-DHED compound. HP guided mouse behavior test. EN assisted in analyzing the data. All authors performed data quantification, discussed the results, and commented on the manuscript.

### Conflict of interest statement

The authors declare that the research was conducted in the absence of any commercial or financial relationships that could be construed as a potential conflict of interest.

## Supplementary material

Supplementary Figure 1 Schematic diagram of the chemical structure of cx-DHED.

Supplementary Figure 2 Effects of cx-DHED on phosphorylated tau in 5xFAD mice. **(A)** Phosphorylated tau (p-Tau) and total tau from whole brain lysates of mice were subjected to western blotting with the AT8 monoclonal antibody. Levels of total tau by immunoblotting with the H150 antibody were examined. **(B)** Quantification of immunoreactivity on AT8 shows elevated tau phosphorylation in 6 months-old Tg mice. **(C)** Quantification of immunoreactivity of total tau reveals no change in all groups. The bar shows the percentage of GAPDH normalized to the density of p-Tau **(B)** and total Tau **(C)** on western blot bands. All data were given as means ± standard error of the mean (SEM) (*N* = 4 mice per group). ^*^*P*<*0.05* compared with WT-v mice, ^#^*P*<*0.05* compared with Tg-v mice.

Supplementary Figure 3 Effects of cx-DHED on learning and memory deficits in 5xFAD mice during the probe test of Morris water maze. **(A)** Schematic drawing of MWM test. Four visual cues were used, and the pool was divided into four quadrants (zone). Plat form marked as PF. **(B)** We did not observe any difference in velocity (swim speed) during the acquisition training. **(C)** In Tg-v, swimming time in target quadrant is reduced compared with WT-v, and is increased in the Tg-DD compared to the Tg-v. **(D)** We did not observe any difference in distance (path length) during probe test between all groups. All data were given as means ± standard error of the mean (SEM) (*N* = 10 mice per group).

Supplementary Figure 4 Effects of cx-DHED on synaptic proteins. Synaptosome fractions of cortical lysates were used to detect loss of synaptic proteins in mice. **(A)** Western blot analysis of GluN1, GluN2B, Rph3A, and RAB3A from cortical synaptosome fractions. **(B–E)** The bar shows the percentage of GAPDH normalized to the density of GluN1 **(B)**, GluN2B **(C)**, Rph3A **(D)**, and RAB3A **(E)** on western blot bands. All data were given as means ± standard error of the mean (SEM) (*N* = 4 mice per group).

Supplementary Table 1 Multiple reaction monitoring parameters for cx-DHED analysis.

## References

[B1] Ahn S. H. Jeon S. H. Tsuruo T. Shim C. K. Chung S. J. (2004). Pharmacokinetic characterization of dehydroevodiamine in the rat brain. J. Pharm. Sci. 93, 283–292. 10.1002/jps.10546 14705186

[B2] Aisen P. S. Saumier D. Briand R. Laurin J. Gervais F. Tremblay P. (2006). A phase II study targeting amyloid-beta with 3APS in mild-to-moderate Alzheimer disease. Neurology 67, 1757–1763. 10.1212/01.wnl.0000244346.08950.64 17082468

[B3] Bilikiewicz A. Gaus W. (2004). Colostrinin (a naturally occurring, proline-rich, polypeptide mixture) in the treatment of Alzheimer's disease. J. Alzheimers Dis. 6, 17–26. 10.3233/JAD-2004-6103 15004324

[B4] Cercato M. C. Vázquez C. A. Kornisiuk E. Aguirre A. I. Colettis N. Snitcofsky M. (2016). GluN1 and GluN2A NMDA receptor subunits increase in the hippocampus during memory consolidation in the rat. Front. Behav. Neurosci. 10:242 10.3389/fnbeh.2016.00242 28133447 PMC5233710

[B5] Cho K. O. Hunt C. A. Kennedy M. B. (1992). The rat brain postsynaptic density fraction contains a homolog of the Drosophila discs-large tumor suppressor protein. Neuron 9, 929–942. 10.1016/0896-6273(92)90245-9 1419001

[B6] Cortese G. P. Burger C. (2016). Neuroinflammatory challenges compromise neuronal function in the aging brain: postoperative cognitive delirium and Alzheimer's disease. Behav Brain Res. 322(Pt B):269–279. 10.1016/j.bbr.2016.08.027 27544872 PMC5450823

[B7] Daulatzai M. A. (2016). Fundamental role of pan-inflammation and oxidative-nitrosative pathways in neuropathogenesis of Alzheimer's disease in focal cerebral ischemic rats. Am. J. Neurodegener. Dis. 5, 102–130. 27335702 PMC4913220

[B8] Decker M. (2005). Novel inhibitors of acetyl- and butyrylcholinesterase derived from the alkaloids dehydroevodiamine and rutaecarpine. Eur. J. Med. Chem. 40, 305–313. 10.1016/j.ejmech.2004.12.003 15725500

[B9] Egan M. F. Kost J. Tariot P. N. Aisen P. S. Cummings J. L. Vellas B. (2018). Randomized trial of verubecestat for mild-to-moderate Alzheimer's disease. N. Engl. J. Med. 378, 1691–1703. 10.1056/NEJMoa1706441 29719179 PMC6776074

[B10] Ferri C. P. Prince M. Brayne C. Brodaty H. Fratiglioni L. Ganguli M. (2005). Global prevalence of dementia: a Delphi consensus study. Lancet 366, 2112–2117. 10.1016/S0140-6736(05)67889-0 16360788 PMC2850264

[B11] Frost B. Jacks R. L. Diamond M. I. (2009). Propagation of tau misfolding from the outside to the inside of a cell. J. Biol. Chem. 284, 12845–12852. 10.1074/jbc.M808759200 19282288 PMC2676015

[B12] Gervais F. Chalifour R. Garceau D. Kong X. Laurin J. Mclaughlin R. (2001). Glycosaminoglycan mimetics: a therapeutic approach to cerebral amyloid angiopathy. Amyloid 8(Suppl. 1), 28–35. 11676287

[B13] Giuffrida M. L. Caraci F. Pignataro B. Cataldo S. De Bona P. Bruno V. (2009). Beta-amyloid monomers are neuroprotective. J. Neurosci. 29, 10582–10587. 10.1523/JNEUROSCI.1736-09.2009 19710311 PMC6665714

[B14] Grosshans D. R. Clayton D. A. Coultrap S. J. Browning M. D. (2002). LTP leads to rapid surface expression of NMDA but not AMPA receptors in adult rat CA1. Nat. Neurosci. 5, 27–33. 10.1038/nn779 11740502

[B15] Guo J. L. Lee V. M. (2011). Seeding of normal Tau by pathological Tau conformers drives pathogenesis of Alzheimer-like tangles. J. Biol. Chem. 286, 15317–15331. 10.1074/jbc.M110.209296 21372138 PMC3083182

[B16] Hardy J. Allsop D. (1991). Amyloid deposition as the central event in the etiology of Alzheimers-disease. Trends Pharmacol. Sci. 12, 383–388. 10.1016/0165-6147(91)90609-V 1763432

[B17] Hoffmann H. Gremme T. Hatt H. Gottmann K. (2000). Synaptic activity-dependent developmental regulation of NMDA receptor subunit expression in cultured neocortical neurons. J. Neurochem. 75, 1590–1599. 10.1046/j.1471-4159.2000.0751590.x 10987840

[B18] Honig L. S. Vellas B. Woodward M. Boada M. Bullock R. Borrie M. (2018). Trial of solanezumab for mild dementia due to Alzheimer's disease. N. Engl. J. Med. 378, 321–330. 10.1056/NEJMoa1705971 29365294

[B19] Kim H. J. Chang K. A. Ha T. Y. Kim J. Ha S. Shin K. Y. (2014a). S100A9 knockout decreases the memory impairment and neuropathology in crossbreed mice of Tg2576 and S100A9 knockout mice model. PLoS ONE 9:e88924 10.1371/journal.pone.0088924 24586443 PMC3934881

[B20] Kim H. J. Shin K. Y. Chang K. A. Ahn S. Choi H. S. Kim H. S. (2014b). Dehydroevodiamine center dot HCl improves stress-induced memory impairments and depression like behavior in rats. Korean J. Physiol. Pharmacol. 18, 55–59. 10.4196/kjpp.2014.18.1.55 24634597 PMC3951824

[B21] Kuperstein I. Broersen K. Benilova I. Rozenski J. Jonckheere W. Debulpaep M. (2010). Neurotoxicity of Alzheimer's disease Abeta peptides is induced by small changes in the Abeta42 to Abeta40 ratio. EMBO J. 29, 3408–3420. 10.1038/emboj.2010.211 20818335 PMC2957213

[B22] La Joie R. Landeau B. Perrotin A. Bejanin A. Egret S. Pélerin A. (2014). Intrinsic connectivity identifies the hippocampus as a main crossroad between Alzheimer's and semantic dementia-targeted networks. Neuron 81, 1417–1428. 10.1016/j.neuron.2014.01.026 24656258

[B23] Monyer H. Burnashev N. Laurie D. J. Sakmann B. Seeburg P. H. (1994). Developmental and regional expression in the rat brain and functional properties of four NMDA receptors. Neuron 12, 529–540. 10.1016/0896-6273(94)90210-0 7512349

[B24] Oakley H. Cole S. L. Logan S. Maus E. Shao P. Craft J. (2006). Intraneuronal beta-amyloid aggregates, neurodegeneration, and neuron loss in transgenic mice with five familial Alzheimer's disease mutations: potential factors in amyloid plaque formation. J. Neurosci. 26, 10129–10140. 10.1523/JNEUROSCI.1202-06.2006 17021169 PMC6674618

[B25] Pannee J. Portelius E. Minthon L. Gobom J. Andreasson U. Zetterberg H. (2016). Reference measurement procedure for CSF amyloid beta (Abeta)1-42 and the CSF Abeta1-42/Abeta1-40 ratio–a cross-validation study against amyloid PET. J. Neurochem. 139, 651–658. 10.1111/jnc.13838 27579672

[B26] Paoletti P. Bellone C. Zhou Q. (2013). NMDA receptor subunit diversity: impact on receptor properties, synaptic plasticity and disease. Nat. Rev. Neurosci. 14, 383–400. 10.1038/nrn3504 23686171

[B27] Park C. H. Kim S. H. Choi W. Lee Y. J. Kim J. S. Kang S. S. (1996). Novel anticholinesterase and antiamnesic activities of dehydroevodiamine, a constituent of *Evodia rutaecarpa*. Planta Med. 62, 405–409. 10.1055/s-2006-957926 8923803

[B28] Park C. H. Lee Y. J. Lee S. H. Choi S. H. Kim H. S. Jeong S. J. (2000). Dehydroevodiamine.HCl prevents impairment of learning and memory and neuronal loss in rat models of cognitive disturbance. J. Neurochem. 74, 244–253. 10.1046/j.1471-4159.2000.0740244.x 10617126

[B29] Parsons M. P. Raymond L. A. (2014). Extrasynaptic NMDA receptor involvement in central nervous system disorders. Neuron 82, 279–293. 10.1016/j.neuron.2014.03.030 24742457

[B30] Peng J. H. Zhang C. E. Wei W. Hong X. P. Pan X. P. Wang J. Z. (2007). Dehydroevodiamine attenuates tau hyperphosphorylation and spatial memory deficit induced by activation of glycogen synthase kinase-3 in rats. Neuropharmacology 52, 1521–1527. 10.1016/j.neuropharm.2007.02.008 17434540

[B31] Rijal Upadhaya A. Capetillo-Zarate E. Kosterin I. Abramowski D. Kumar S. Yamaguchi H. (2012). Dispersible amyloid beta-protein oligomers, protofibrils, and fibrils represent diffusible but not soluble aggregates: their role in neurodegeneration in amyloid precursor protein (APP) transgenic mice. Neurobiol. Aging 33, 2641–2660. 10.1016/j.neurobiolaging.2011.12.032 22305478

[B32] Sans N. Petralia R. S. Wang Y. X. Blahos J. II. Hell J. W. Wenthold R. J. (2000). A developmental change in NMDA receptor-associated proteins at hippocampal synapses. J. Neurosci. 20, 1260–1271. 10.1523/JNEUROSCI.20-03-01260.2000 10648730 PMC6774158

[B33] Santa-Maria I. Hernández F. Del Rio J. Moreno F. J. Avila J. (2007). Tramiprosate, a drug of potential interest for the treatment of Alzheimer's disease, promotes an abnormal aggregation of tau. Mol. Neurodegener. 2:17 10.1186/1750-1326-2-17 17822548 PMC2048960

[B34] Sanz-Clemente A. Nicoll R. A. Roche K. W. (2013). Diversity in NMDA receptor composition: many regulators, many consequences. Neuroscientist 19, 62–75. 10.1177/1073858411435129 22343826 PMC3567917

[B35] Sauvee M. Didierlaurent G. Latarche C. Escanye M. C. Olivier J. L. Malaplate-Armand C. (2014). Additional use of Abeta(4)(2)/Abeta(4)(0) ratio with cerebrospinal fluid biomarkers P-tau and Abeta(4)(2) increases the level of evidence of Alzheimer's disease pathophysiological process in routine practice. J. Alzheimers Dis. 41, 377–386. 10.3233/JAD-131838 24614902

[B36] Selkoe D. J. (2011). Alzheimer's disease. Cold Spring Harb. Perspect. Biol. 3:a004457 10.1101/cshperspect.a004457 21576255 PMC3119915

[B37] Shankar G. M. Li S. Mehta T. H. Garcia-Munoz A. Shepardson N. E. Smith I. (2008). Amyloid-beta protein dimers isolated directly from Alzheimer's brains impair synaptic plasticity and memory. Nat. Med. 14, 837–842. 10.1038/nm1782 18568035 PMC2772133

[B38] Shin K. Y. Kim K. Y. Suh Y. H. (2017). Dehydroevodiamine.HCl enhances cognitive function in memory-impaired rat models. Korean J. Physiol. Pharmacol. 21, 55–64. 10.4196/kjpp.2017.21.1.55 28066141 PMC5214911

[B39] Stanic J. Carta M. Eberini I. Pelucchi S. Marcello E. Genazzani A. A. (2015). Rabphilin 3A retains NMDA receptors at synaptic sites through interaction with GluN2A/PSD-95 complex. Nat. Commun. 6:10181 10.1038/ncomms10181 26679993 PMC4703873

[B40] Tan M. G. Chua W. T. Esiri M. M. Smith A. D. Vinters H. V. Lai M. K. (2010). Genome wide profiling of altered gene expression in the neocortex of Alzheimer's disease. J. Neurosci. Res. 88, 1157–1169. 10.1002/jnr.22290 19937809

[B41] Tohda C. (2016). New age therapy for Alzheimer's disease by neuronal network reconstruction. Biol. Pharm. Bull. 39, 1569–1575. 10.1248/bpb.b16-00438 27725432

[B42] Townsend M. Cleary J. P. Mehta T. Hofmeister J. Lesne S. O'hare E. (2006). Orally available compound prevents deficits in memory caused by the Alzheimer amyloid-beta oligomers. Ann. Neurol. 60, 668–676. 10.1002/ana.21051 17192927

[B43] Unsworth W. P. Kitsiou C. Taylor R. J. (2013). An expedient protecting-group-free total synthesis of (+/-)-dievodiamine. Org. Lett. 15, 3302–3305. 10.1021/ol4013469 23786450

[B44] Van Cauwenberghe C. Van Broeckhoven C. Sleegers K. (2016). The genetic landscape of Alzheimer disease: clinical implications and perspectives. Genet. Med. 18, 421–430. 10.1038/gim.2015.117 26312828 PMC4857183

[B45] Webster S. J. Bachstetter A. D. Nelson P. T. Schmitt F. A. Van Eldik L. J. (2014). Using mice to model Alzheimer's dementia: an overview of the clinical disease and the preclinical behavioral changes in 10 mouse models. Front. Genet. 5:88 10.3389/fgene.2014.00088 24795750 PMC4005958

[B46] Wilcox K. C. Lacor P. N. Pitt J. Klein W. L. (2011). Abeta oligomer-induced synapse degeneration in Alzheimer's disease. Cell. Mol. Neurobiol. 31, 939–948. 10.1007/s10571-011-9691-4 21538118 PMC3146579

[B47] Wortmann M. (2012). Dementia: a global health priority–highlights from an ADI and World Health Organization report. Alzheimers Res. Ther. 4:40 10.1186/alzrt143 22995353 PMC3580397

