# Supplementary figures and images for: Effects of a Dehydroevodiamine-Derivative on Synaptic Destabilization and Memory Impairment in the 5xFAD, Alzheimer's Disease Mouse Model
Source: Front Behav Neurosci. 2018 Nov 13;12:273. doi: 10.3389/fnbeh.2018.00273 (PMC6243640; doi:10.3389/fnbeh.2018.00273)

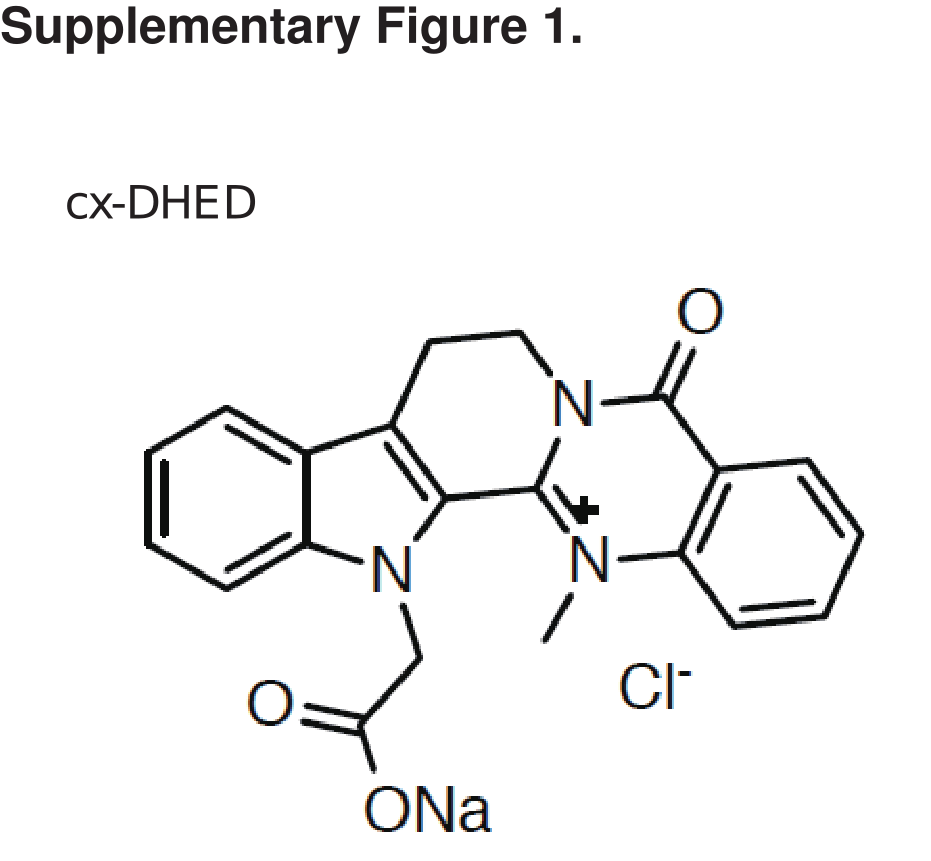

Supplement: Supplementary Figure 1 — Schematic diagram of the chemical structure of cx-DHED. [file Image_1.tiff]

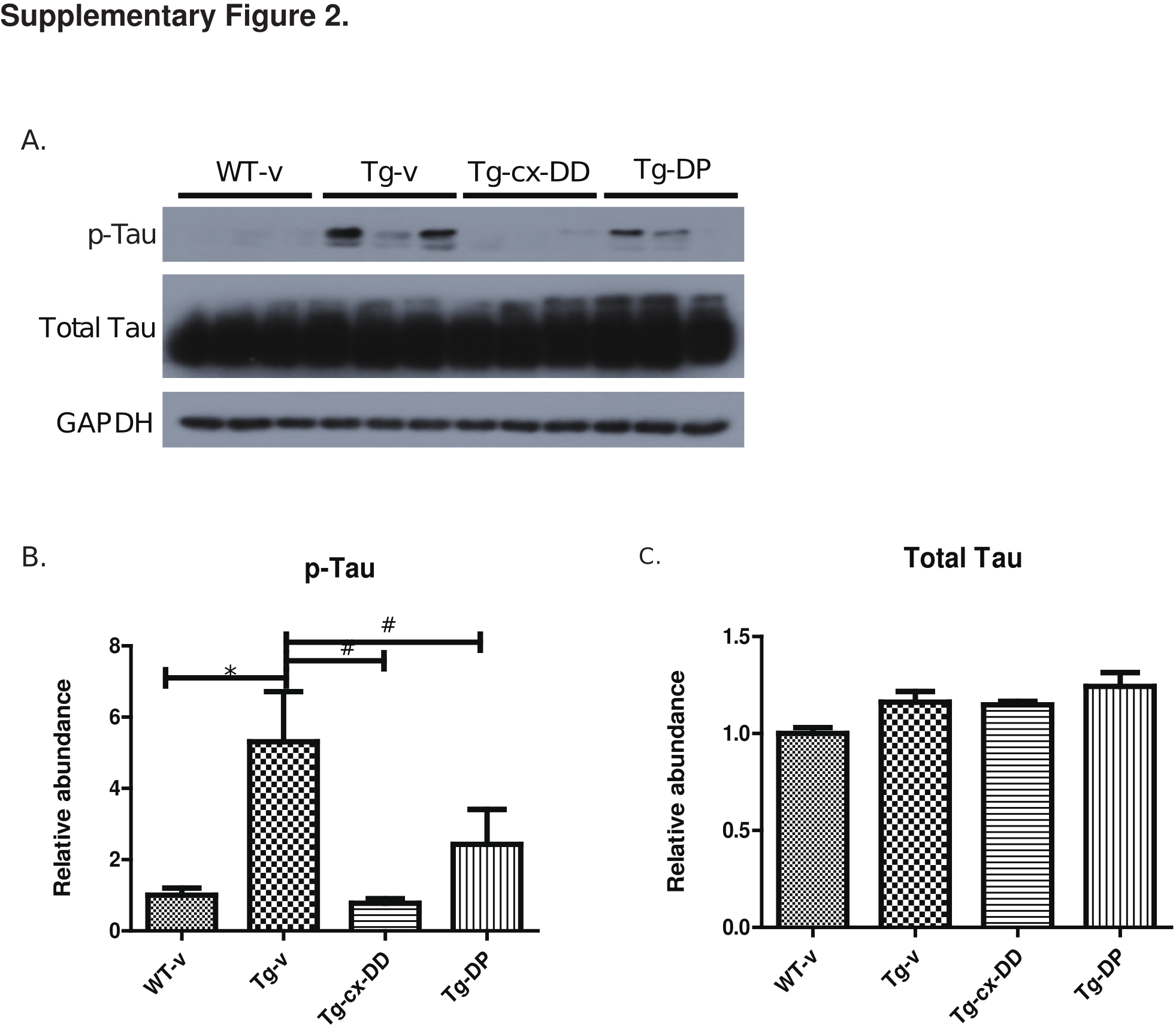

Supplement: Supplementary Figure 2 — Effects of cx-DHED on phosphorylated tau in 5xFAD mice. (A) Phosphorylated tau (p-Tau) and total tau from whole brain lysates of mice were subjected to western blotting with the AT8 monoclonal antibody. Levels of total tau by immunoblotting with the H150 antibody were examined. (B) Quantification of immunoreactivity on AT8 shows elevated tau phosphorylation in 6 months-old Tg mice. (C) Quantification of immunoreactivity of total tau reveals no change in all groups. The bar shows the percentage of GAPDH normalized to the density of p-Tau (B) and total Tau (C) on western blot bands. All data were given as means ± standard error of the mean (SEM) (N = 4 mice per group). *P<0.05 compared with WT-v mice, #P<0.05 compared with Tg-v mice. [file Image_2.tiff]

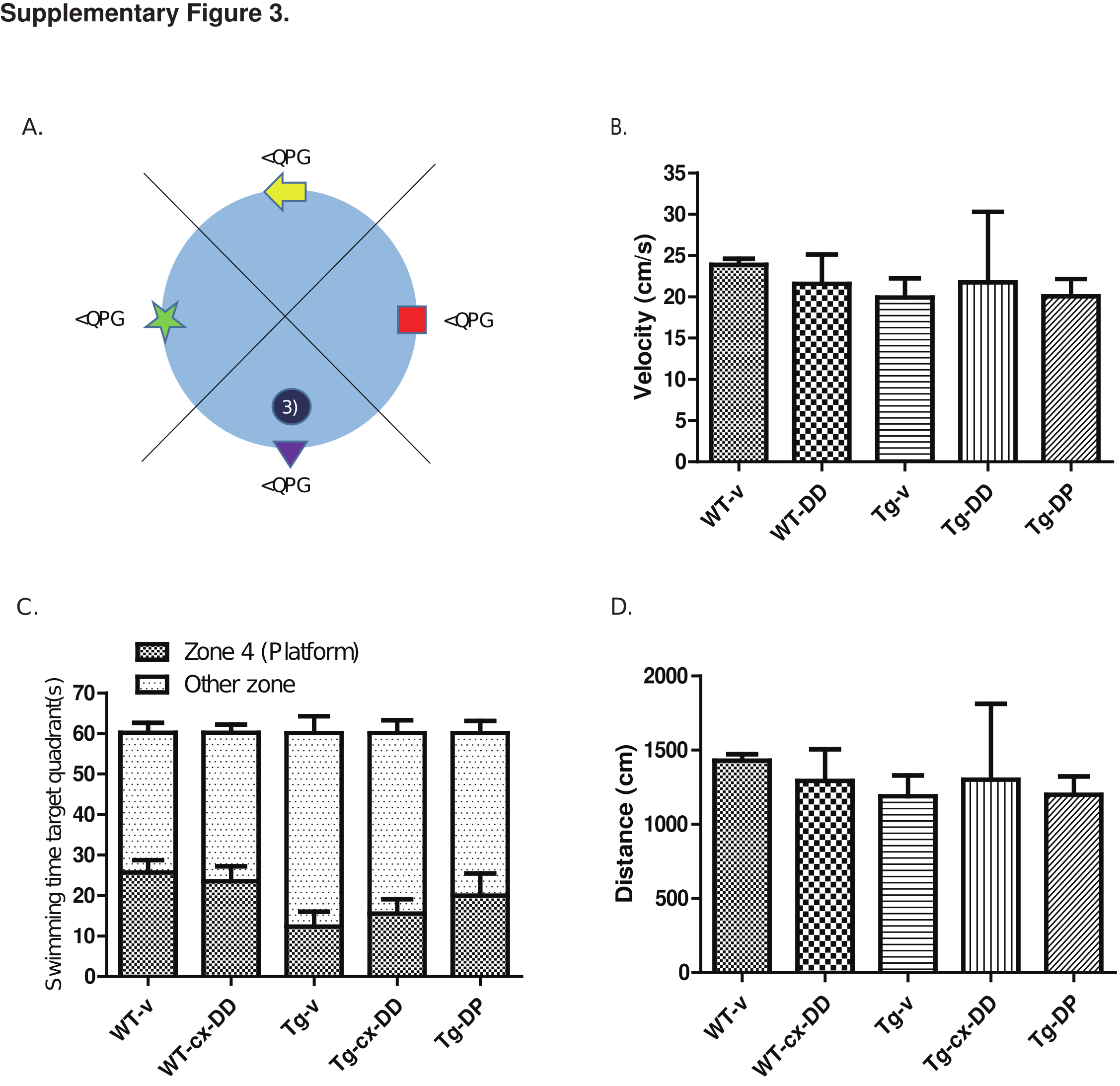

Supplement: Supplementary Figure 3 — Effects of cx-DHED on learning and memory deficits in 5xFAD mice during the probe test of Morris water maze. (A) Schematic drawing of MWM test. Four visual cues were used, and the pool was divided into four quadrants (zone). Plat form marked as PF. (B) We did not observe any difference in velocity (swim speed) during the acquisition training. (C) In Tg-v, swimming time in target quadrant is reduced compared with WT-v, and is increased in the Tg-DD compared to the Tg-v. (D) We did not observe any difference in distance (path length) during probe test between all groups. All data were given as means ± standard error of the mean (SEM) (N = 10 mice per group). [file Image_3.tiff]

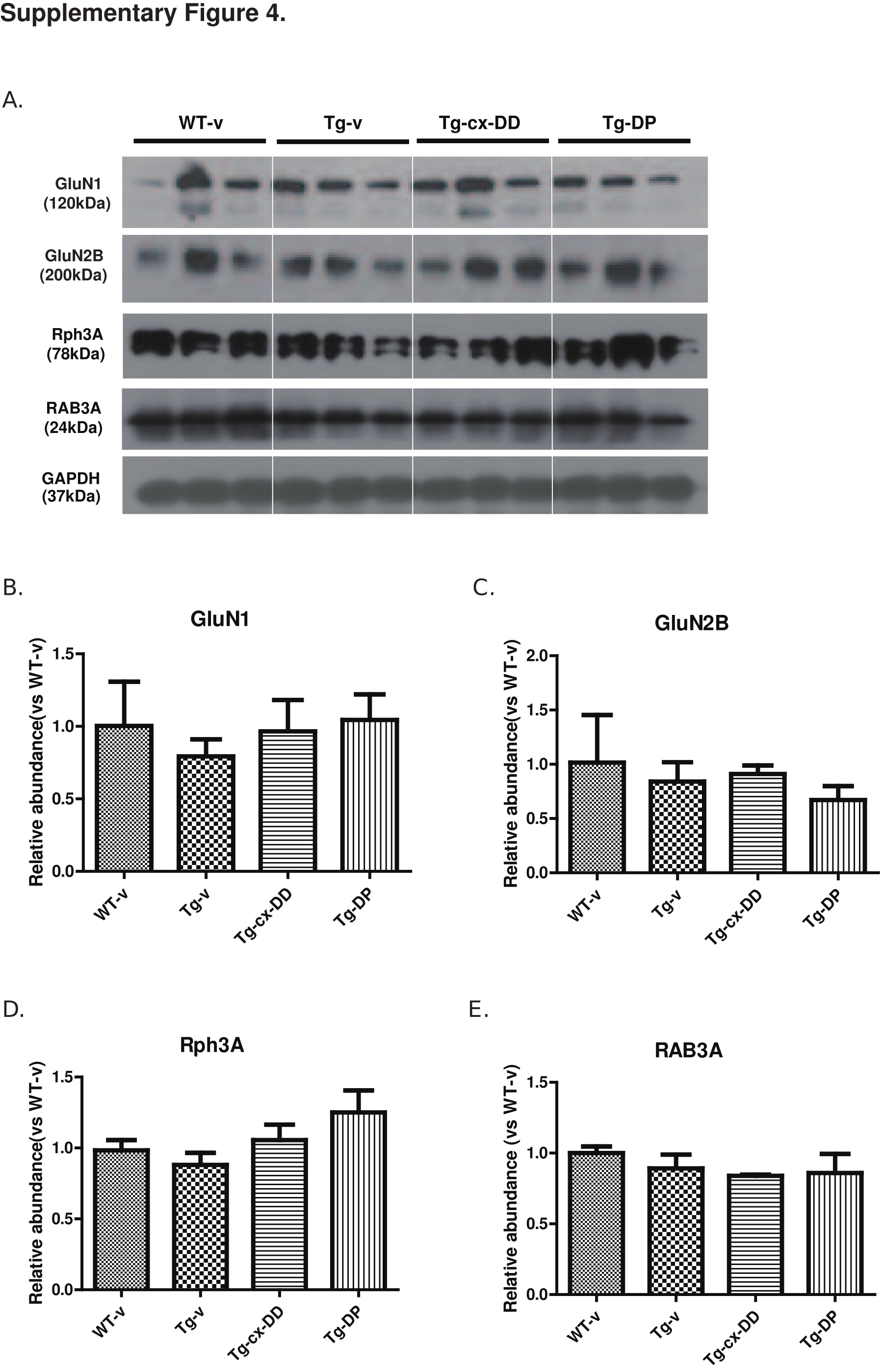

Supplement: Supplementary Figure 4 — Effects of cx-DHED on synaptic proteins. Synaptosome fractions of cortical lysates were used to detect loss of synaptic proteins in mice. (A) Western blot analysis of GluN1, GluN2B, Rph3A, and RAB3A from cortical synaptosome fractions. (B–E) The bar shows the percentage of GAPDH normalized to the density of GluN1 (B), GluN2B (C), Rph3A (D), and RAB3A (E) on western blot bands. All data were given as means ± standard error of the mean (SEM) (N = 4 mice per group). [file Image_4.tiff]
