# Supplementary material for: Effects of a Dehydroevodiamine-Derivative on Synaptic Destabilization and Memory Impairment in the 5xFAD, Alzheimer's Disease Mouse Model
Source: Front Behav Neurosci. 2018 Nov 13;12:273. doi: 10.3389/fnbeh.2018.00273 (PMC6243640; doi:10.3389/fnbeh.2018.00273)
Supplement: Supplementary Table 1 — Multiple reaction monitoring parameters for cx-DHED analysis. [file Table_1.docx]

**Supplementary Table 1. Multiple reaction monitoring parameters for cx-DHED analysis**

|  | Precursor ion (*m/z*) | Product ion (*m/z*) | Fragmentor | Collision energy |
| --- | --- | --- | --- | --- |
| Quantifier | 360.2 | 300.2 | 200 | 40 |
| Qualifier | 360.2 | 272.2 | 150 | 40 |
